# Exploring the in vivo digestion of plant proteins in broiler chickens

**DOI:** 10.3382/ps/pew444

**Published:** 2017-02-23

**Authors:** E. Recoules, H. Sabboh-Jourdan, A. Narcy, M. Lessire, G. Harichaux, V. Labas, M.J. Duclos, S. Réhault-Godbert

**Affiliations:** * INRA, UR83 Poultry Research Unit, 37380 Nouzilly, France; † INRA, Plat-form for Integrative Analysis of Biomolecules and Phenomic of Animals of Bio-agronomic Interest, 37380 Nouzilly, France; ‡ INRA, UMR INRA 85, Physiology of Reproduction and Behaviors, 37380 Nouzilly, France; § CNRS, UMR 7247, 37380 Nouzilly, France; # University of François Rabelais, 37000 Tours, France

**Keywords:** chicken, protein, digestion, proteomic

## Abstract

The use of various protein sources (industry by-products, proteaginous) in poultry diets requires a greater understanding of protein digestion mechanisms. The aim of this study was to characterize the molecular actors required for protein digestion in broilers fed 4 different diets containing soybean meal, rapeseed meal, pea, or corn distiller's dried grain with solubles as the only protein source. The digesta of the digestive tract segments were collected and soluble proteins were analyzed by SDS-PAGE. SDS-PAGE analyses revealed 5 ubiquitous bands in digesta of all digestive tract segments regardless of the diet, whereas 3 bands were diet-specific. The digesta of the jejunum were further submitted to proteomic analysis. Forty-two proteins of chicken origin and 17 plant proteins were identified in digesta samples by mass spectrometry. Fifteen proteins of chicken origin were specific to one diet and 18 were common to all diets. By homology with mammals, these proteins are thought to be involved in protein, lipid, carbohydrate, and nucleic acid metabolism and also in intestinal homeostasis. Some of the 17 plant proteins were found to be not fully digested (soybean meal, rapeseed meal, and pea diets) and others were identified as protease inhibitors (soybean meal and pea diets). This study provides a comprehensive analysis of the physiological proteins involved in the digestion of 4 protein sources used in broiler diets. Such an approach, combined with the analysis of insoluble components of these different protein sources, would contribute to define whether these protein sources could be more largely used in poultry nutrition. It also would allow identifying ways to improve their digestibility in broiler chickens (feed additives such as exogenous proteases or processing to inactivate anti-nutritional factors, for instance).

## INTRODUCTION

The constant increase in demand for poultry meat by consumers requires an increase in production capacity. When supplemented with free methionine, soybean meal is the main protein source in poultry nutrition as it is well balanced in amino acids and provides suitable amounts of digestible amino acids (Stein et al., 2008). Soybean meal for animal feeds is mainly imported from the United States, Brazil, and Argentina (Martin, 2014). In countries that are currently not self-sufficient in proteins, the use of soybean meal in broiler diets is challenged because of price volatility, genetically modified origin, and environmental impact due to transportation and deforestation (de Visser et al., 2014). The gap between local supply and demand for the commonly used feedstuffs for animal production is expected to increase over the forthcoming decades, supporting the need to evaluate the usefulness of locally available, unconventional feedstuffs in feed formulations (Ravindran, 2010). However, nutritional values differ among feedstuffs, some of them being better digested and utilized by animals. The challenge is thus to improve the digestive efficiency of these various feedstuffs without affecting performance levels. Digestive efficiency of various protein sources has been enhanced by reducing anti-nutritional factor levels through varietal selection (Slominski et al., 1999), technological processing (Quinsac et al., 1994; Mińkowski, 2002), and by increasing the accessibility of nutrients by the addition of exogenous enzymes (Adeola and Cowieson, 2011; Freitas et al., 2011). However, although the basics of digestive physiology in poultry were described in the 1980s (Sklan and Hurwitz, 1980; Larbier and Leclercq, 1992; Klasing, 1999), there is very little information concerning the precise mechanisms of the process of feed digestion throughout the digestive tract. Considering the diversity of protein feedstuffs, it is important to better understand which specific factors affect feedstuff efficiency. Previous experiments have contributed to unravel digestive mechanisms involved in the digestion of pea proteins in pigs (mass spectrometry; Le Gall et al., 2005) and broilers (SDS-PAGE and immunohistochemistry; Crevieu et al., 1997a; Crevieu et al., 1997b; Gabriel et al., 2008). These studies have highlighted the benefits of using biochemical/proteomic techniques to study protein digestion in animals. Farm animal proteomics has been essentially developed for calf and swine and results revealed its importance to understand the complex biological systems that control animal physiology and pathology (Bendixen et al., 2011). A similar approach applied to a chicken model seems to be particularly relevant with regard to the increasing amount of data available in databanks, thanks to the sequencing and annotation of the chicken genome, and the concomitant development of bioinformatics to predict protein functions. In this context, we used proteomic tools to further analyze protein digestion in chicken. The soluble proteins of the digesta collected throughout the digestive tract of broilers fed 4 different protein sources were analyzed by SDS-PAGE, and a proteomic analysis of the jejunal digesta (containing proventriculus, pancreatic, and intestinal secretions) was performed. Soluble proteins were investigated to evaluate 1) which enzymes and proteins are endogenously secreted during the digestion process, and 2) which dietary proteins resist proteolysis, including anti-nutritional factors. The focus on soluble proteins would facilitate the identification of endogenous proteins participating in the digestion process and of dietary proteins that are partially digested, or that are solubilized but that resist proteolysis, up to that stage of digestion. This study will contribute to a better understanding of protein digestion mechanisms in broilers and will help to define new strategies to enhance intrinsic digestibility of alternative protein sources in birds.

## MATERIALS AND METHODS

Procedures involving the use of birds were approved by the regional Ethics Committee (Approval N°C37-175-1). All experiments were conducted according to the European legislation on the “protection of animals used for experimental and other scientific purposes” set by the European community Council Directive of November 24, 1986 (86/609/ECC).

### Experimental Design and Sample Collection

Ross PM3 broiler chicks (n = 72) were purchased from a commercial hatchery (Grelier, Saint-Laurent-de-la-Plaine, France). From day 1 to 7, chicks were housed 2 birds per cage to avoid social isolation and were fed a standard starter diet (NRC, 1994). On day 7, chicks were allocated to one of the 4 experimental diets based on body weight (n = 18 per group) in order to have 4 experimental groups with balanced body weight. They were housed in individual cages (one bird per cage) until day 21. Semi-synthetic experimental diets were formulated (Table 1). The protein fraction was based on a single protein source: soybean meal (**S**), rapeseed meal (**R**), pea (**P**), or corn distiller's dried grains with solubles (**C**). Between day 7 and 17, the experimental diet was supplied as a 50/50 mix with the starter diet. Between day 17 and 21, birds were fed the experimental diet. Diets were pelleted at a temperature of 55 °C for the S, P, and C diets and 60 °C for the R diet. Water (2%) was added for the pelleting process. Pelleted diets (2.5 mm in diameter) and tap water were provided ad libitum. Birds were kept under 22 h of light from day 1 to 3 and then under 18 h of light until day 21.

**Table 1.** Composition of experimental diets

	S^1^	P	C	R
Ingredients (g/kg)				
Soybean meal	350			
Pea		850		
Corn distiller's dried grain with solubles			630	
Rapeseed meal				300
Corn starch	391.2	57.9	204.5	424.5
Sucrose	195.6	28.9	102.3	212.3
Soybean oil	30	30	30	30
Dicalcium phosphate	15	15	15	15
Calcium carbonate	10	10	10	10
Salt	3	3	3	3
Vitamin-mineral premix^2^	5	5	5	5
Clinacox anticoccidian^3^	0.2	0.2	0.2	0.2
**Estimated nutritional value**				
Metabolizable energy (kcal/kg)	3,130	2,920	2,780	3,000
Crude protein (g/kg)	156	167	172	146
**Measured nutritional value**				
Dry matter (%)	92.2	90.5	93.6	91.9
Cell wall (% dry matter)	6.8	10.9	18.8	11.2
Crude protein (g/kg)	143	169	192	97
Lys	0.94	1.23	0.67	0.51
Thr	0.62	0.64	0.74	0.43
Meth	0.195	0.161	0.406	0.181
Cystine + Cystéine	0.205	0.242	0.329	0.208
Meth + Cys	0.400	0.403	0.735	0.389
Trp	0.208	0.157	0.210	0.121
Val	0.74	0.77	0.95	0.49
Ile	0.71	0.68	0.74	0.37
Leu	1.19	1.17	1.79	0.66
Arg	1.07	1.31	1.13	0.52
Phe	0.80	0.79	0.92	0.38
Tyr	0.48	0.54	0.66	0.26
His	0.39	0.40	0.49	0.25
Ser	0.810	0.80	0.92	0.43
Ala	0.68	0.71	1.15	0.42
Aspartic acid	1.74	1.86	1.47	0.69
Glutamic acid	2.78	2.68	3.47	1.57
Gly	0.67	0.72	0.92	0.48
Pro	0.79	0.67	1.22	0.60
Trypsin inhibitor (ITU^4^/mg)	3.4	6.6	-	-

^1^Diets: S, soybean meal; P, pea; C, corn distiller's dried grain with solubles; R, rapeseed meal.

^2^Premix: 5.2 mg/15 000 IU of vitamin A (retinyl acetate), 0.108 mg/ 4 300 IU of vitamin D3 (cholecalciferol), 91 IU of vitamin E (dl-α-tocopherol), 5 mg of vitamin K3 (menadione), 5 mg of vitamin B1 (thiamine), 8 mg of vitamin B2 (riboflavin), 100 mg of vitamin B3 (PP, niacin), 25 mg of vitamin B5 (Ca panthotenate), 7 mg of vitamin B6 (pyridoxine), 0.3 mg of vitamin B8 (biotin, H), 3 mg of vitamin B9 (folic acid), 0.02 mg of vitamin B12 (cyanocobalamin), 550 mg of choline, 60 mg of Fe (FeSO4), 20 mg of Cu (CuSO4), 80 mg of Mn (MnO), 90 mg of Zn (ZnSO4), 0.6 mg of Co (CoSO4), 2 mg of I [Ca(IO3)2], 0.2 mg of Se (Na2SeO3), 1.18 g of Ca (CaCO3).

^3^Active ingredients: diclazuril 0.2%.

^4^ITU: International trypsin unit.

On day 21, all birds were individually weighed after 5 hours of fasting. After synchronized feeding (one bird fed every 5 min for 3 h), birds were euthanized by a lethal injection of sodium pentobarbital (one mL per kg of body weight) in the wing vein. Fasting and synchronized feeding were required 1) to ensure the presence of digesta in different segments of the digestive tract, 2) to have a similar feed intake among birds, and 3) to limit inter-individual variability due to differences in digestion time. Digesta from the different parts of the digestive tract (proventriculus/gizzard, duodenum, jejunum, and ileum) were collected by gentle squeezing. It is noteworthy that this method can introduce some proteins from the intestinal wall and mucus in the digesta, but it was preferred to water flushing to avoid any dilution of the samples and to be able to measure the protein concentration. Of the 18 birds per diet, 6 were randomly selected for pH measurements and the remaining 12 were used for protein analyses. The pH of digesta was measured with a SevenGo^™^ SG2 pH-meter and InLabbs Solids Pro electrode (Mettler Toledo, Viroflay, France). All samples were stored at - 80 °C until further analysis.

### Statistical Analysis

Statistical analyses were performed with R software. The Shapiro-Wilk test was used to test the normality of data. Within each digestive tract segment, the effect of the diet on the pH of digesta was tested with a GLM or a non-parametric Kruskal-Wallis test depending on the data distribution. A difference was considered significant at *P* < 0.05. Data are expressed as means ± sd of 6 independent samples for each diet. Different letters denote values that are significantly different (*P* < 0.05) among diets, within the same digestive tract segment.

### Buffering Capacity of the Diets

The buffering capacity of the diets was measured according to the method described by Lawlor et al (2005).

### Sample Preparation for Protein Analyses

The protein source on one hand and crude digesta on the other hand were homogenized in 0.5 M Tris-HCl buffer (pH 8.8 (diet) or 6.8 (digesta), 150 mM NaCl) for 30 s on ice using a T25 Ultra Turrax (IKA, Staufen, Germany) disperser (one g of diet/10 mL of buffer; 2 g of digesta/4 mL of buffer) and then centrifuged at 4,000 rpm for 10 min at 4 °C. Protein concentration in the supernatant was determined using the Dc-Biorad Assay (Bio-Rad, Marnes-la-Coquette, France), with bovine serum albumin (Interchim, Montluçon, France) as the standard. An initial set of individual analyses allowed checking the homogeneity of the electrophoresis profile between birds within each diet and each digestive tract segment (data not shown). Then the supernatants were pooled by digestive tract segment (12 birds per treatment) to assess the mean response of birds to a specific diet. The pools were constituted according to the individual protein concentration so that the quantity of each sample included in the pool was inversely proportional to its protein concentration. The samples were stored at – 20 °C until further analysis.

### Electrophoretic Analysis

Soluble proteins present in the digesta were analyzed by 12.5% SDS-PAGE (Laemmli, 1970). Molecular weight standards (Precision Plus Protein^™^ All Blue Standards, #161.0373, BioRad, Marnes-la-Coquette, France) and samples corresponding to pooled supernatants (40 μg of protein) were loaded on a 12.5% running gel (one mm, 15 wells). At the end of migration, proteins were stained with Coomassie blue.

### Gel and Liquid Chromatography – Tandem Mass Spectrometry (GeLC-MS/MS) Analyses

Major bands from blue-stained SDS-PAGE gels from the jejunum were excised, in-gel digested by trypsin, and analyzed by nano LC-MS/MS. All experiments were performed on a LTQ Orbitrap Velos Mass Spectrometer (Thermo Fisher Scientific, Bremen, Germany) coupled to an Ultimate 3000 RSLC chromatographer (Dionex, Amsterdam, The Netherlands). Samples were loaded on an LCPackings trap column (Acclaim PepMap 100 C18, 100 mm i.d6 2 cm long, 3 mm particles) and desalted for 10 min at 5 mL/min with solvent A. Mobile phases consisted of (A) 0.1% formic acid, 97.9% water, and 2% acetonitrile, and (B) 0.1% formic acid, 15.9% water, and 84% acetonitrile. Separation was conducted using an LCPackings nano-column (Acclaim PepMap C18, 75 mm i.d6 50 cm long, 3 mm particles) at 300 nl/min by applying a gradient consisting of 4–55% B for 60 min. The mass spectrometer was operated in data-dependent scan mode. Full scan MS spectra surveys (from 300 to 1,800 mass to charge ratio, **m/z**) were acquired in the Orbitrap analyzer with R = 60,000. The 20 most intense ions with charge states ≥2 were sequentially isolated (isolation width, 2 m/z; one microscan) and fragmented in the high-pressure linear ion trap by low-energy collision-induced dissociation with normalized collision energy of 35% and wideband-activation enabled. Dynamic exclusion was active for 30 s with a repeat count of one. Polydimethylcyclosiloxane (m/z, 445.1200025) ions were used for internal calibration. MS/MS ion searches were performed using Mascot search engine v 2.2 (Matrix Science, London, UK) against the chordata, plant, and procaryote sections of a locally maintained copy of non-redundant protein sequences-National Center for Biotechnology Information (**nr-NCBI**), downloaded 01/22/2014). The parameters used for database searches included trypsin as a protease with 2 missed cleavages allowed, and carbamidomethylcysteine, oxidation of methionine, and N-terminal protein acetylation as variable modifications. The tolerance of the ions was set at 5 ppm for parent and 0.8 Da for fragment ion matches. Mascot results obtained from the target and decoy database searches were subjected to Scaffold 3 software (v 3.6, Proteome Software, Portland, Oregon). Peptide and protein identification was performed by the Peptide and Protein Prophet algorithms (Keller et al., 2002; Nesvizhskii et al., 2003) with a probability of 95%. Only proteins identified with at least 2 exclusive peptides were considered from the mass spectrometry results. Proteins were classified according to their functional information described in the UniProtKB database (http://www.uniprot.org/) or by homology with other species.

## RESULTS

### Birds’ Performance Levels

The aim of the study was to explore protein digestion in chickens using various protein sources in the diet. The formulations were made trying to balance the diet, without altering feed intake, knowing that nutritional values and anti-nutritional factors can be different among diets and can subsequently affect digestibility and birds’ performances. Birds’ performances are reported in Supporting Material 1.

### Buffering Capacity of the Diets.

The buffering capacity of the diets was 163, 165, 123, and 214 for the S, R, P, and C diets, respectively.

### pH of the Digesta

**Figure 1.**
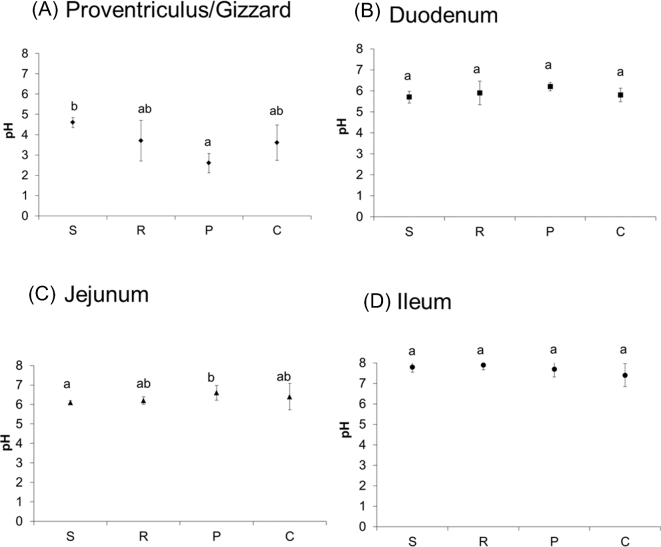
pH in the digesta of birds fed soybean meal (S, n = 6), rapeseed meal (R, n = 6), pea (P, n = 5), or corn distiller's dried grains with solubles (C, n = 6). Data are expressed as means ± sd of 6 independent samples for each diet. Different letters denote values that are significantly different (*P* < 0.05) among diets, within the same digestive tract segment.

The pH values of the digesta for each diet and within each digestive tract segment are reported in Figure 1. The average pH increased from 3.6 (proventriculus/gizzard) to 7.7 (ileum). The diet had a significant effect on the pH only in the proventriculus/gizzard and the jejunum. In the proventriculus/gizzard, the S diet had the highest pH (4.6 ± 0.25) and the P diet the lowest (2.6 ± 0.47). The pH values of the R and C diets were intermediate. In the jejunum, the trend was reversed, with the lowest value for the S diet (6.1 ± 0.10) and the highest value for the P diet (6.6 ± 0.38). In contrast, in the duodenum and the ileum the diet had no effect on the pH value.

### SDS-PAGE Analysis of Digesta (proventriculus/gizzard, duodenum, jejunum, ileum)

**Figure 2.**
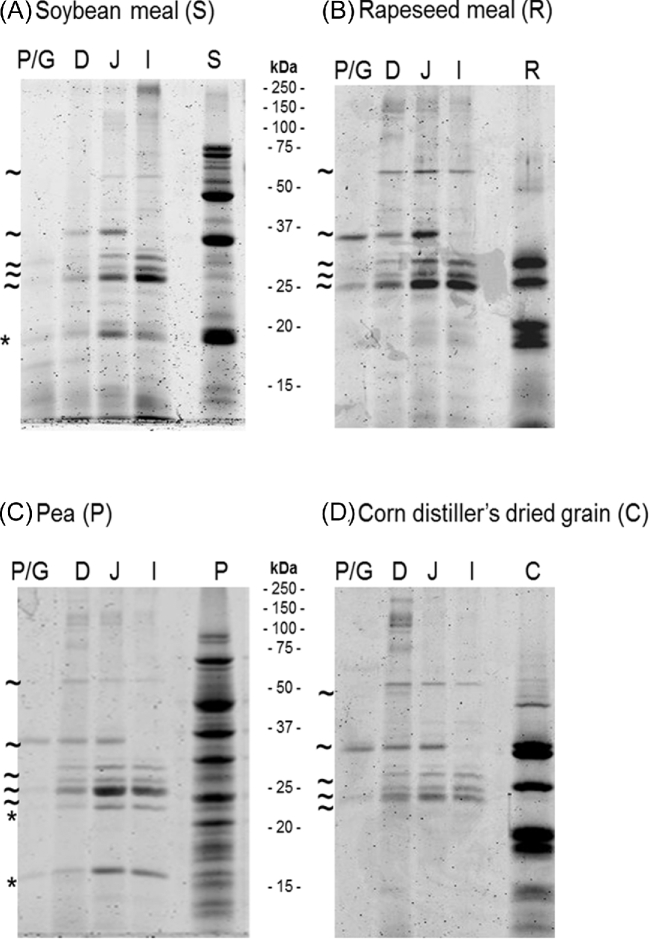
SDS-PAGE of the digesta of the different parts of the digestive tract (P/G: proventriculus/gizzard; D: duodenum; J: jejunum; I: ileum, n = 12 per diet) compared to the raw protein source (S: soybean meal; R: rapeseed meal; P: pea; C: corn distiller's dried grain with solubles). The tilde represents bands that are present in all diets and the asterisk represents bands that are diet-specific.

The results of SDS-PAGE analyses performed on pooled samples are presented in Figure 2. The SDS-PAGE profiles for the 4 protein sources were different from each other. However, the bands observed in the digesta were similar among diets. Five bands (25, 26, 27, 36, and 55 kDa) were present in all samples, regardless of the diet (Figures 2A, 2B, 2C, and 2D). In contrast, one band of 18 kDa for the S diet (Figure 2A) and 2 bands of 16 and 24 kDa for the P diet (Figure 2C) appeared to be diet-specific. Moreover, the protein profiles of the proventriculus/gizzard contained slightly detectable bands for all diets. Some bands that were still visible in the digesta of the anterior parts of the intestine (duodenum, jejunum) were absent in the ileal digesta (36 kDa bands, Figures 2A, 2B, 2C and 2D).

### Protein Identification in the Jejunum

In the present study, the focus was on the jejunum, as it is at the intermediate stage of the digestion process. Focusing on the jejunum provided identification of 1) proteins partially hydrolyzed at the proventriculus and duodenum levels, 2) endogenous enzymes secreted by the proventriculus and the pancreas, and 3) endogenous proteins that will disappear (be absorbed) in the ileum, after physiological activation in the anterior parts of the intestine. Analyses in the jejunum therefore provided a snapshot of the molecular actors required to digest dietary proteins.

The 55, 36, 27, 26, 25, 24, 18, and 16 kDa bands from the jejunum were analyzed by GeLC-MS/MS. A total of 67 different proteins were identified, with 50 in the *Gallus gallus* chordate and 17 in the plant chordate. No significant proteins were identified when the data were submitted to bacteria taxonomy (NCBInr_220114 database, containing 23,073,856 proteins in the bacteria databank). Of the 50 *Gallus gallus* proteins, 6 were immunoglobulins (Ig lambda chain V-1 region precursor [Gallus gallus], gi|513788266; Ig light chain precursor V-J region - chicken (fragment), gi|104728; immunoglobulin alpha heavy chain [Gallus gallus], gi|5705960; immunoglobulin heavy chain variable region [Gallus gallus], gi|161513287; immunoglobulin lambda chain variable region [Gallus gallus], gi|1536800; immunoglobulin light-chain VJ region, partial [Gallus gallus], gi|212164). Since the presence of immunoglobulins varied considerably from one individual to another, they are not discussed further in this article. Similarly, two “low quality” proteins possibly associated with defects in the genome assembly and/or model as predicted by NCBI, were not taken into consideration, as their presence might be controversial (uncharacterized protein LOC423943 [Gallus gallus], gi|513192591; uncharacterized protein LOC424904 [Gallus gallus], gi|513200420). The analysis was performed on the 59 remaining proteins. The results for the S, R, P, and C diets are presented in Tables 2, 3, 4 and 5, respectively. More information about the identification of proteins is provided in Supporting Material 2. Of these 59 proteins, 12 were described in the NCBI databank as proteins predicted by automated computational analysis using genomic sequence (NW_003764144.1) annotated using the gene prediction method Gnomon, and supported by mRNA and EST evidence. Proteomic analysis confirmed the presence of these proteins in the digestive tract, knowing that 2 exclusive peptides are sufficient to identify a protein without ambiguity.

**Table 2.** Proteins identified in the jejunal digesta of birds fed the soybean meal (S) diet

Proteins identified	Accession number	Gene ID	MW^1^	SDS-PAGE bands
			(kDa)	(kDa)
Gallus gallus proteins				
*Protein digestion and regulation*				
Chymotrypsinogen 2-like precursor	gi|478621048	431235	29	18, 25, 26, 27, 55
Chymotrypsin-like elastase family member 2A precursor	gi|157817197	419486	29	18, 25, 26, 27
Chymotrypsin-like elastase family member 1 precursor	gi|157073915	425200	28	25
Trypsinogen	gi|25814806	396344/396345	27	18, 25, 26, 27, 55
Pepsin A precursor	gi|45382405	395691	42	18, 25, 26, 27, 55
PREDICTED: Proproteinase E-like	gi|363742042	100857116	22	18, 25, 26, 27
Carboxypeptidase A1 preproprotein	gi|45383013	395276	47	18, 25, 26, 27
Carboxypeptidase A2 precursor	gi|380748957	416682	47	18, 25, 26
Carboxypeptidase A5 precursor	gi|356582409	416683	47	18, 25, 26, 27
Carboxypeptidase B preproprotein	gi|476007880	424888	47	25, 26, 27
Chymotrypsin-C	gi|483968280	430670	29	25, 26
Aminopeptidase N	gi|272753691	395667	109	25, 26
Xaa-Pro dipeptidase	gi|119331076	415776	55	25, 26, 55
Aspartyl aminopeptidase, partial [Columba livia]^2^	gi|449275360	-	43	25
Meprin A subunit alpha precursor^2^	gi|478733046	422060	80	55
PREDICTED: Probable aminopeptidase^2^	gi|118100855	419314	56	25
PREDICTED: Leukocyte elastase inhibitor-like [Meleagris gallopavo]^2^	gi|326917097	100545931	43	25
*Carbohydrate metabolism*				
PREDICTED: Maltase-glucoamylase, intestinal	gi|513200006	425007	210	18, 25, 26, 27, 55
PREDICTED: Maltase-glucoamylase, intestinal-like isoform X4	gi|513157898	417691	76	18, 25, 26, 27, 55
Amylase, alpha 2A; pancreatic precursor	gi|377520154	414140	57	25, 26, 27, 55
Triosephosphate isomerase^2^	gi|45382061	-	27	25
*Nucleic acid metabolism*				
Cytidine deaminase precursor	gi|45384384	395773	31	25, 26
K123 protein precursor	gi|45384326	395873	31	55
*Intestinal homeostasis*				
PREDICTED: Angiopoietin-related protein 1-like	gi|363745669	417459	39	25, 26, 27, 55
PREDICTED: Fibrinogen-like protein A-like, partial	gi|513235012	100857922	26	26
PREDICTED: Gamma-glutamyltranspeptidase 1^2^	gi|118098729	416945	62	55
Hydroxyacylglutathione hydrolase, mitochondrial precursor	gi|61098280	416537	34	26
PREDICTED: Phospholipid scramblase 2 isoform 1 ^2^	gi|118095086	424883	34	25
PREDICTED: Deleted in malignant brain tumors 1 protein-like	gi|513233665	426826	63	55
PREDICTED: Intestinal-type alkaline phosphatase-like	gi|363737177	100859876	56	26, 27, 55
Acidic chitinase precursor^2^	gi|45383307	395072	52	26
Angiotensin-converting enzyme ^2^	gi|268370291	419953	147	55
Angiotensin-converting enzyme 2 ^2^	gi|118084115	418623	93	55
*Lipid metabolism*				
PREDICTED: Neutral ceramidase-like ^2^	gi|513190876	100540641		25
Pancreatic triacylglycerol lipase precursor ^2^	gi|475506556	423916	51	25
Phospholipase A2 group IB precursor	gi|224458364	416980	34	55
**Soybean proteins**				
RecName: Full = Glycinin G1	gi|121276	-	56	18, 25, 26, 27, 55
Glycinin A5A4B3 subunit [Glycine soja]	gi|126144648	-	64	18, 25, 26, 27, 55
Glycinin A3B4 subunit [Glycine soja]	gi|126144646	-	58	18, 25
Glycinin G2 precursor [Glycine max]	gi|351725363	547900	54	18, 26
Glycinin G3 precursor - soybean	gi|99909	-	54	18
Beta-conglycinin alpha subunit [Glycine max]	gi|335353923	-	70	25, 26, 27
Prepro beta-conglycinin alpha prime subunit [Glycine max]	gi|32328882	-	72	18, 25, 27, 55
Beta-conglycinin beta subunit [Glycine max]	gi|341603993	-	50	18, 25, 26, 27, 55
Chain A, soybean agglutinin complexed with 2,6-pentasaccharide	gi|6729836	-	28	25, 26, 27
Kunitz trypsin inhibitor [Glycine max]	gi|13375349	-	24	18, 25
7S seed globulin precursor [Glycine max]	gi|1401240	-	46	26

^1^MW: theoretical molecular weight.

^2^Proteins specific to the soybean meal diet.

**Table 3.** Proteins identified in the jejunal digesta of birds fed the rapeseed meal (R) diet

Proteins identified	Accession number	Gene ID	MW^1^	SDS-PAGE bands
			(kDa)	(kDa)
Gallus gallus proteins				
*Protein digestion and regulation*				
Chymotrypsinogen 2-like precursor	gi|478621048	431235	29	25, 26, 27
Chymotrypsin-like elastase family member 2A precursor	gi|157817197	419486	29	25, 26, 27
Trypsinogen	gi|25814806	396344/396345	27	25, 26, 27, 36, 55
Pepsin A precursor	gi|45382405	395691	42	25, 26, 27, 36, 55
PREDICTED: Proproteinase E-like	gi|363742042	100857116	22	25, 26, 27
Carboxypeptidase A2 precursor	gi|380748957	416682	47	25
Carboxypeptidase A5 precursor	gi|356582409	416683	47	25
Carboxypeptidase B preproprotein	gi|476007880	424888	47	27
Chymotrypsin-C	gi|483968280	430670	29	25, 26
Xaa-Pro dipeptidase	gi|119331076	415776	55	25
*Carbohydrate metabolism*				
PREDICTED: Maltase-glucoamylase, intestinal	gi|513200006	425007	210	26, 55
PREDICTED: Maltase-glucoamylase, intestinal-like isoform X4	gi|513157898	417691	76	36, 55
Amylase, alpha 2A; pancreatic precursor	gi|377520154	414140	55	55
*Nucleic acid metabolism*				
K123 protein precursor	gi|45384326	395873	31	55
*Intestinal homeostasis*				
PREDICTED: Deleted in malignant brain tumors 1 protein-like	gi|513233665	426826	63	27, 36
Hydroxyacylglutathione hydrolase, mitochondrial precursor	gi|61098280	416537	34	26
PREDICTED: Intestinal-type alkaline phosphatase-like	gi|363737177	100859876	56	55
Serpin B6	gi|57530448	420895	43	25, 27
Desmoplakin I [Homo sapiens]	gi|1147813	-	332	26
Rapeseed proteins		-		
Cruciferin subunit [Brassica napus]	gi|12751302	-	54	25, 26, 27, 36
Cruciferin cru4 subunit [Brassica napus]	gi|17805		46	25, 26, 27, 36

^1^MW: theoretical molecular weight.

**Table 4.** Proteins identified in the jejunal digesta of birds fed the pea (P) diet

Proteins identified	Accession number	Gene ID	MW^1^ (kDa)	SDS-PAGE bands
				
Gallus gallus proteins				
*Protein digestion and regulation*				
Chymotrypsinogen 2-like precursor	gi|478621048	431235	29	16, 24, 25, 26, 27, 36, 55
Chymotrypsin-like elastase family member 2A precursor	gi|157817197	419486	29	16, 24, 25, 26, 27
Chymotrypsin-like elastase family member 1 precursor	gi|157073915	425200	28	16, 24
Trypsinogen	gi|25814806	396344/396345	27	16, 24, 25, 26, 27, 36
Trypsin I-P1 precursor ^2^	gi|45382399	431235	26	24, 25
Pepsin A precursor	gi|45382405	395691	42	16, 24, 25, 26, 27, 36
PREDICTED: Proproteinase E-like	gi|363742042	100857116	22	25, 26, 36
Carboxypeptidase A1 preproprotein	gi|45383013	395276	47	16, 24, 25, 26, 27, 36
Carboxypeptidase A2 precursor	gi|380748957	416682	47	16, 24, 25, 26, 36
Carboxypeptidase A5 precursor	gi|356582409	416683	47	16, 24, 25, 26, 27, 36
Carboxypeptidase B preproprotein	gi|476007880	424888	47	16, 24, 25, 26, 27, 36
Chymotrypsin-C	gi|483968280	430670	29	25, 26
Aminopeptidase N	gi|272753691	395667	109	25, 26
Xaa-Pro dipeptidase	gi|119331076	415776	55	24, 25, 26, 55
Latexin ^2^	gi|477507250	771519	25	26
*Carbohydrate metabolism*				
PREDICTED: Maltase-glucoamylase, intestinal	gi|513200006	425007	210	25, 26, 36, 55
PREDICTED: Maltase-glucoamylase, intestinal-like isoform X4	gi|513157898	417691	76	16, 25, 26, 36
Amylase, alpha 2A; pancreatic precursor	gi|377520154	414140	57	25, 26, 55
*Nucleic acid metabolism*				
Cytidine deaminase precursor	gi|45384384	395773	31	25, 26
*Intestinal homeostasis*				
Hydroxyacylglutathione hydrolase, mitochondrial precursor	gi|61098280	416537	34	25, 26
Superoxide dismutase [Cu-Zn] ^2^	gi|45384218	395938	15.7	16
*Lipid metabolism*				
Phospholipase A2 group IB precursor	gi|224458364	416980	17	36
Pea proteins				
Chain A, X-ray crystal structure of a pea lectin-trimannoside complex	gi|443232	-	20	16, 24, 25
Chain A, crystal structure of a Bowman-Birk inhibitor from pea seeds	gi|4389007	-	8	16
Convicilin [Pisum sativum]	gi|7339551	-	72	25
RecName: Full = legumin A2	gi|126161	-	59	16

^1^MW: theoretical molecular weight.

^2^Proteins specific to the pea diet.

**Table 5.** Proteins identified in the jejunal digesta of birds fed the corn distillers’ dried grain with solubles (C) diet

Proteins identified	Accession number	Gene ID	MW^1^	SDS-PAGE bands
			(kDa)	
Gallus gallus proteins				
*Protein digestion and regulation*				
Chymotrypsinogen 2-like precursor	gi|478621048	431235	29	25, 26, 27, 36
Chymotrypsin-like elastase family member 2A precursor	gi|157817197	419486	29	25, 26, 27
Trypsinogen	gi|25814806	396344/396345	27	25, 26, 27, 36
Pepsin A precursor	gi|45382405	395691	42	25, 26, 27, 36, 55
PREDICTED: Proproteinase E-like	gi|363742042	100857116	22	25, 26, 27
Carboxypeptidase A1 preproprotein	gi|45383013	395276	47	27
Carboxypeptidase A2 precursor	gi|380748957	416682	47	25
Carboxypeptidase A5 precursor	gi|356582409	416683	47	25, 27, 36
Carboxypeptidase B preproprotein	gi|476007880	424888	47	25, 27
Chymotrypsin-C	gi|483968280	430670	29	27
Aminopeptidase N	gi|272753691	395667	109	25, 27
Xaa-Pro dipeptidase	gi|119331076	415776	55	25, 27, 55
*Carbohydrate metabolism*				
PREDICTED: Maltase-glucoamylase, intestinal	gi|513200006	425007	210	25, 27, 36, 55
PREDICTED: Maltase-glucoamylase, intestinal-like isoform X4	gi|513157898	417691	76	27
Amylase, alpha 2A; pancreatic precursor	gi|377520154	414140	57	25, 27, 55
*Nucleic acid metabolism*				
Cytidine deaminase precursor	gi|45384384	395773	31	27
*Intestinal homeostasis*				
PREDICTED: Angiopoietin-related protein 1-like	gi|363745669	417459	39	36
PREDICTED: Fibrinogen-like protein A-like, partial	gi|513235012	100857922	26	27
Serpin B6	gi|57530448	420895	43	25

^1^MW: theoretical molecular weight.

No corn proteins were identified in the study.

Forty-seven proteins were identified in the digesta of chickens fed the S diet (Table 2), 36 being *Gallus gallus* proteins and 11 being soybean proteins *(Glycine max)*. Eight proteases, 8 peptidases, and one protease inhibitor were involved in protein digestion. Four proteins were identified as enzymes with a predicted role in carbohydrate metabolism. Two proteins are related to nucleic acid metabolism and 3 to lipid metabolism, and we found 10 proteins that could have a role in maintaining intestinal homeostasis. Eleven soybean proteins were identified with several glycinin subunits, conglycinins, agglutinin, a globulin, and a trypsin inhibitor (Kunitz trypsin inhibitor). The diet-specific band (18 kDa) seemed to concentrate several glycinin- and conglycinin-derived proteins, suggesting greater resistance of these proteins to digestive enzymes compared to other plant proteins. The major protein of this fraction was the Kunitz trypsin inhibitor (emPAI = 4.89), followed by Glycinin G1 (emPAI = 1.29).

Twenty-one proteins were identified in the digesta of chickens fed the R diet (Table 3), 19 being *Gallus gallus* proteins and 2 being rapeseed proteins (*Brassica napus*). Six proteases and 4 peptidases are involved in rapeseed protein digestion. Three proteins are related to carbohydrate metabolism, one to nucleic acid metabolism, and 5 may be involved in maintaining intestinal homeostasis. The 2 rapeseed proteins were cruciferin subunits. All bands seemed common to all other diets, and all the proteins of chicken origin identified by GeLC-MS/MS also were identified in the other diets except for desmoplakin, which was identified in the 26 kDa band as a very minor component (emPAI 0.0281). Desmoplakin is a key protein involved in cell-cell adhesion and is present in all organisms (Bornslaeger et al., 1996). It also was identified in the S and P diets but with only one peptide. We therefore did not consider it was specific to the R diet and it is not discussed further.

Twenty-six different proteins were identified in the digesta of chickens fed the P diet (Table 4), including 22 *Gallus gallus* and 4 pea proteins (*Pisum sativatum)*. Fifteen proteins corresponding to 8 proteases, 6 peptidases, and one protease inhibitor participated in protein digestion. Two maltase glucoamylases and amylase alpha 2 A are defined as major actors of carbohydrate metabolism, one protein is involved in nucleic acid metabolism, and 2 proteins may participate in intestinal homeostasis. We also identified one protein with a potential role in lipid metabolism. Four pea proteins corresponding to lectin, a trypsin inhibitor (Bowman-Birk inhibitor, **BBI**), convicilin, and legumin A2 were identified. Interestingly, plant proteins were identified in the 2 diet-specific bands (16 and 24 kDa): 1) lectin with emPAI of 12.4 (16 kDa) and 0.87 (24 kDa), 2) BBI with emPAI of 0.86 (16 kDa), and 3) legumin A2 (16 kDa). Endogenous proteins involved in protein digestion and regulation also were identified in these 2 specific bands and they were similar to proteins found in bands of 25, 26, 27, 36, and 55 kDa in other diets. This suggests a possible interaction between these *Gallus gallus* proteins and some components of the P diet. These results and the fact that bands of 16 and 24 kDa were still visible in the ileum (Figure 2C) suggest that these pea proteins are particularly resistant to digestion.

Nineteen *Gallus gallus* proteins were identified in the digesta of chickens fed the C diet (Table 5). These consisted of 12 proteins related to protein digestion, including 6 proteases and 6 peptidases, 3 proteins associated with carbohydrate metabolism, one with nucleic acid metabolism, and 3 that could participate in intestinal homeostasis. No corn protein could be identified, although the databank related to corn contained about 175,000 protein sequences when the analysis was performed.

## DISCUSSION

We analyzed the key factors and molecules (pH, digestive enzymes, and proteins) in dietary protein digestion in response to 4 different protein sources to evaluate the molecular mechanisms that trigger protein digestion in chickens.

Protein digestion may be affected by the non-protein part of the protein sources and of the diet (fiber content, anti-nutritional factors; Smits and Annison, 1996; Vadivel and Pugalenthi, 2010). This non-protein part can explain differences in the mechanisms involved in protein digestion throughout the digestive tract. Therefore, to better appreciate the specific effect of the protein sources on digestion, diets were formulated with similar ingredients (except the protein source). Only the amounts of starch and sucrose differed between diets, which would have a very low impact on digestion since these components are very well digested in birds, especially as we used purified starch.

The pH value of the digesta was lower in the proventriculus/gizzard for the P diet. The hypothesis to explain this difference in proventriculus/gizzard pH among diets is the buffering capacity of the diet (capacity to resist acidic or alkaline environments). The buffering effects of pea and soybean meal in broilers have been reported to be 121 and 276, respectively (Gomez and Corniaux, 2009). In our study, the results also indicated that the P diet had the lowest buffering capacity, which might explain why birds fed the P diet had a lower pH in the proventriculus/gizzard. The pH in the digestive tract segments is very important in protein digestion as it affects the processing of endogenous proteases into their mature form, but also the solubility of the dietary proteins.

Using a proteomic approach, we identified proteins of chicken origin and enzymes that were present up to that point of the digestive tract, and also dietary proteins that were resistant to digestive proteolysis.

Overall, we identified 59 different proteins, which were then classified using functional information available in UniProtKB and homology to other species, in order to predict their function in digestion. Moreover, we confirmed the presence of 12 proteins that were still considered as “predicted proteins” in databanks (angiopoietin-related protein 1-like [Gallus gallus], gi|363745669; deleted in malignant brain tumors 1 protein-like [Gallus gallus], gi|513233665; fibrinogen-like protein A-like, partial [Gallus gallus], gi|513235012; gamma-glutamyltranspeptidase 1 [Gallus gallus], gi|118098729; intestinal-type alkaline phosphatase-like [Gallus gallus], gi|363737177; leukocyte elastase inhibitor-like [Meleagris gallopavo], gi|326917097; maltase-glucoamylase, intestinal [Gallus gallus], gi|513200006; maltase-glucoamylase, intestinal-like isoform X4 [Gallus gallus], gi|513157898; neutral ceramidase [Gallus gallus], gi|513190876; phospholipid scramblase 2 isoform 1 [Gallus gallus], gi|118095086; probable aminopeptidase NPEPL1 [Gallus gallus], gi|118100855; proproteinase E-like [Gallus gallus], gi|363742042).

The present study proposes an overview of the major proteins involved in chicken protein digestion and of the specific features induced by dietary proteins. Among all the proteins identified, 18 were found in all diets and 15 were diet-specific. This section is subdivided into 3 parts: 1) proteins common to all diets, 2) diet-specific proteins, and 3) plant proteins that were partially or totally resistant to hydrolysis.

Of the 18 common proteins, 12 were involved in protein digestion and regulation (essentially enzymes), 3 in carbohydrate metabolism, one in nucleic acid metabolism, and 2 in intestinal homeostasis.

**Figure 3.**
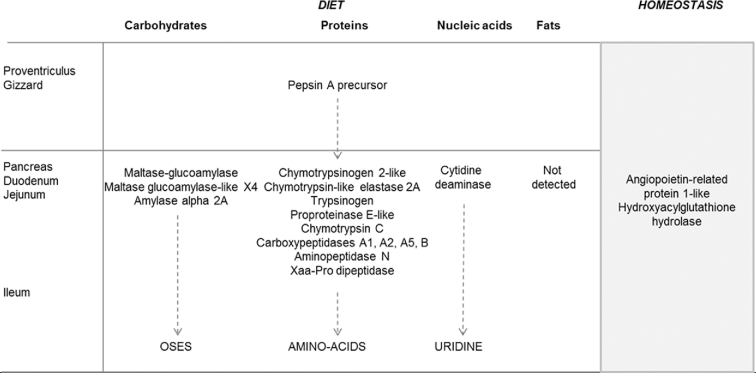
Schematic representation of the main molecular actors involved in protein digestion in broilers throughout the digestive tract. This representation is proposed based on common proteins found in the jejunal digesta of birds fed soybean meal, rapeseed meal, pea, or corn distillers’ dried grains with solubles. Their role in the digestive process is proposed based on knowledge available in literature and by homology with mammals.

Pepsin is a proteolytic enzyme that is secreted as a precursor called pepsinogen. It is released by the chief cells in the proventriculus, auto-activates in an acidic environment, and degrades dietary proteins into peptides. Pepsin is a universal protease in vertebrates. However, compared to other species, chicken pepsin remains stable at slightly alkaline pH, suggesting that it might still participate in protein digestion in the lower part of the gastrointestinal tract (BaudyŠ and Kostka, 1983). Trypsinogen is secreted by the pancreas and needs to be activated by enterokinases (Wang et al., 1995) located in the brush border of enterocytes. Once activated, trypsin activates other proteases and peptidases secreted as zymogens by the pancreas. In ruminants, trypsin activates carboxypeptidase A1 precursor, proproteinase E, and chymotrypsinogen-C that are secreted by the pancreas as a ternary complex (Kobayashi et al., 1981; Michon et al., 1991; Gomis-Rüth et al., 1997). The active site of the carboxypeptidase A1 precursor is inside the complex and therefore protected from activation by trypsin. Chymotrypsinogen-C and proprotein E have their active sites exposed on the surface of the ternary complex (Michon et al., 1991; Gomis-Rüth et al., 1997). The successive activation of chymotrypsinogen-C and proprotein E by trypsin leads to the release of the carboxypeptidase A1 precursor that is then activated. The complex and the sequential activation may have 2 major objectives: 1) to protect the carboxypeptidase A1 precursor from denaturation in the duodenum because it has to be active in the terminal steps of the digestive process, and 2) to control the mechanism of hydrolysis as chymotrypsin-C and proteinase E (endopeptidases) act before carboxypeptidase A1 (Gomis-Rüth et al., 1997). In rats, Xaa-Pro dipeptidase (also called Prolidase) is a cytosolic exopeptidase that has an important role in protein metabolism and recycling of amino acids from endogenous proteins (Hu et al., 2003). The amylase α 2A pancreatic precursor is secreted by the pancreas as a zymogen and once activated hydrolyses starch. As reported in humans, intestinal maltase-glucoamylase is not secreted by the pancreas but is expressed in the small intestine. It hydrolyses maltose but also can hydrolyse starch when the amylase activity is reduced (Lentze, 1995). In humans, angiopoietin-related protein 1-like and hydroxyacylglutathione hydrolase are not specific to digestion and might have a role in maintaining homeostasis (Cordell et al., 2004; Brindle et al., 2006). Based on the 18 common proteins identified in this study and on information available in the literature, a schematic representation of the main molecular actors involved in protein digestion throughout the digestive tract in chickens is proposed in Figure 3.

In addition to these common proteins, 15 diet-specific proteins were identified (12 in S and 3 in P). Four of the 12 proteins specific to the S diet are involved in protein digestion and regulation, one in carbohydrate metabolism (triosephosphate isomerase), 2 in lipid metabolism (PREDICTED: neutral ceramidase-like, pancreatic triacylglycerol lipase precursor) and 5 in intestinal homeostasis. Interestingly, one of the 4 proteins involved in protein digestion and regulation has been described as an inhibitor of elastase (PREDICTED: leucocyte elastase inhibitor-like), which may have a role in inhibiting chymotrypsin-like elastases 2A and 1. These also were observed in the jejunum of the birds fed the S diet (Table 2) and chymotrypsin-like elastase family member 1 seems specific to the pancreas (Willmann et al., 2016). The 3 others are proteolytic enzymes (aspartyl aminopeptidase, meprin A subunit alpha precursor and PREDICTED: probable aminopeptidase NPEPL1). The high number of *Gallus gallus* proteins identified for this S diet (36) as well as the number of diet-specific proteins (12) suggest specific activation of digestive proteins in response to this protein source. The stimulation of the digestive tract by soybean also could explain the expression of the specific proteins predicted to participate in maintaining intestinal homeostasis (PREDICTED: gamma-glutamyltranspeptidase 1, PREDICTED: phospholipid scramblase 2 isoform 1, acidic chitinase precursor, angiotensin-converting enzyme, and angiotensin-converting enzyme 2). The 3 proteins specific to the P diet seem to be involved in inflammation. Indeed, latexin has a role in immune defense, and expression of the latexin gene is regulated by several proinflammatory stimuli (Aagaard et al., 2005). Superoxide dismutase is present in all organisms and is well known for its role in antioxidant defense in cells exposed to superoxide radicals. Finally, trypsin I-P1 has been reported to have immunological activities in addition to its digestive role in the chicken (Wang et al., 1995). All these specific proteins may have been present in the other diets but in lower amounts and thus may not have been detected by mass spectrometry. The presence of undigested proteins and anti-nutritional factors including trypsin inhibitors in pea and soybean diets may induce an inflammatory situation, resulting in over-expression of proteins involved in intestinal homeostasis.

The analysis of the soluble jejunal digesta provided an overview of the endogenous proteins that trigger digestion in chickens, and also some dietary proteins that resist proteolysis. It is noteworthy that only a few plant proteins were identified in this approach (11 in S, 2 in R, 4 in P, 0 in C). This could be due to proteins that were recovered in the insoluble phase (that were not analyzed in this work) or to proteins that could not be identified because they are not yet referenced in databases. Indeed, at the time we did the analysis, wide differences in data availability were noticed between feed sources. The protein sequences contained in the NCBI databank were 3,689; 18,460; 105,112, and 174,719 for Pisum (pea), Brassica (rapeseed), Glycine (soybean), and Zea (corn), respectively. Despite this, this study provides interesting information on proteins that resist proteolysis and on anti-nutritional factors that could affect the overall digestibility of dietary proteins by directly interfering with digestive protease activity. The BBI found in the jejunum of birds fed the P diet indicates that this inhibitor resists proteolytic degradation in the digestive tract. This is in agreement with previous in vitro observations in which the BBI was found to be resistant to gastric juices and to various proteolytic enzymes (Huisman and Jansman, 1991; Perrot, 1994). Pea legumin (59 kDa) was observed in the 16 kDa band, which indicates that it had been partially hydrolysed into smaller peptides, as previously reported (Perrot, 1994). Similarly, pea convicilin (72 kDa), which has high homology with vicilin (Tzitzikas et al., 2005), was identified in the 25 kDa band, which agrees with previous results (Perrot, 1994) in which the author reported many 25 kDa polypeptides after vicilin digestion by trypsin. Lectin is resistant to pepsin and trypsin hydrolysis (Perrot, 1994). Indeed, we identified this protein in 16, 24, and 25 kDa bands, i.e., relatively close to the theoretical molecular weight (20 kDa), which corroborates previous results found in pigs (Le Gall et al., 2005).

The trypsin inhibitor activity measured in the S diet was 3.4 **TIU**/mg (trypsin inhibitor units), which is in the range of published values (Clarke and Wiseman, 2005; Ravindran et al., 2014). However, the Kunitz trypsin inhibitor (24 kDa) was identified in the 18 and 25 kDa bands, which indicates that it had not or had been partially hydrolyzed. When denatured, this inhibitor is readily digestible by pepsin, chymotrypsin, or trypsin (Kunitz, 1947). However, it seems that this trypsin inhibitor is an unusually stable protein and resists denaturation by heating to 60 °C (Roychaudhuri et al., 2003). Its resistance to thermal denaturation and its ability to be renatured after heating (Roychaudhuri et al., 2004) could explain why it remained detectable at the jejunal level in digesta of broilers. Other soybean proteins such as glycinin and conglycinin were still present in the jejunum and seemed to be partially digested, which is consistent with previous results in which partially digested β-conglycinin polypeptides were observed in the ileal digesta of pigs (Salgado et al., 2003) and glycinin was observed in the ileal digesta of calves (Lallès et al., 1999).

This study provides a comprehensive analysis of the molecular actors involved in protein digestion in broilers using a proteomic approach. It has to be complemented by the proteomic analysis of the digesta in the other digestive tract segments to have a clearer picture of the kinetic of protein digestion and of the overall digestibility of feed by broilers. Interestingly, this study revealed that most proteins identified in the broiler digestive tract are already known proteases or enzymes that have been described in mammalian species, but identified for the first time in birds in this study. Altogether, these findings support the interest of using proteomic approaches to decipher the mechanisms of protein digestion in chickens. In parallel, analysis of the insoluble fraction would provide interesting information to identify which dietary proteins resist digestion, to further develop strategies to improve their digestibility (process to inactivate anti-nutritional factors, exogenous proteases, or varietal selection of plants, for instance).
